# Proteomic analysis of early-stage incompatible and compatible interactions between grapevine and *P. viticola*

**DOI:** 10.1038/s41438-021-00533-y

**Published:** 2021-05-01

**Authors:** Guo-Tian Liu, Bian-Bian Wang, David Lecourieux, Mei-Jie Li, Ming-Bo Liu, Rui-Qi Liu, Bo-Xing Shang, Xiao Yin, Li-Jun Wang, Fatma Lecourieux, Yan Xu

**Affiliations:** 1 State Key Laboratory of Crop Stress Biology for Arid Areas, College of Horticulture, Northwest A&F University, Yangling, China; 2 Key Laboratory of Horticultural Plant Biology and Germplasm Innovation in Northwest China, Ministry of Agriculture, Northwest A&F University, Yangling, China; 3 UMR1287 EGFV, CNRS, Université de Bordeaux, INRAE, Bordeaux Sciences Agro, ISVV, Villenave d’Ornon, France; 4 Institute of Botany, The Chinese Academy of Sciences, Beijing, China

**Keywords:** Biotic, Proteomics

## Abstract

Wild grapevines can show strong resistance to the downy mildew pathogen *P. viticola*, but the associated mechanisms are poorly described, especially at early stages of infection. Here, we performed comparative proteomic analyses of grapevine leaves from the resistant genotype *V. davidii* “LiuBa-8” (LB) and susceptible *V. vinifera* “Pinot Noir” (PN) 12 h after inoculation with *P. viticola*. By employing the iTRAQ technique, a total of 444 and 349 differentially expressed proteins (DEPs) were identified in LB and PN, respectively. The majority of these DEPs were related to photosynthesis, respiration, cell wall modification, protein metabolism, stress, and redox homeostasis. Compared with PN, LB showed fewer downregulated proteins associated with photosynthesis and more upregulated proteins associated with metabolism. At least a subset of PR proteins (PR10.2 and PR10.3) was upregulated upon inoculation in both genotypes, whereas HSP (HSP70.2 and HSP90.6) and cell wall-related XTH and BXL1 proteins were specifically upregulated in LB and PN, respectively. In the incompatible interaction, ROS signaling was evident by the accumulation of H_2_O_2_, and multiple APX and GST proteins were upregulated. These DEPs may play crucial roles in the grapevine response to downy mildew. Our results provide new insights into molecular events associated with downy mildew resistance in grapevine, which may be exploited to develop novel protection strategies against this disease.

## Introduction

Grapevines (*Vitis*) include some of the most widely cultivated and most economically important fruit crops worldwide. Grapevine downy mildew is one of the most devastating oomycete diseases of grapevine. Its causal agent, *Plasmopara viticola* (Berk and Curt) Berl and de Toni, is a strictly obligate biotrophic pathogen that obtains nutrients from living cells of hosts to complete its life cycle through specialized structures called haustoria. It can infect leaves, shoots, tendrils, inflorescences, and young berries and spread into mature berries through rachis infection.

Plants have evolved sophisticated surveillance systems to defend against pathogen attack^1,2^. These may include preformed constitutive barriers such as a strong cell wall, a thickened waxy layer and dense trichomes on the epidermis, and the presence of antimicrobial toxins in the cell vacuole. In addition, plants are able to activate molecular defense pathways upon contact with pathogens. Such induced defense reactions can provoke pathogen-associated molecular patterns (PAMPs) leading to PAMP-triggered immunity (PTI). However, some pathogens may counter this defense by releasing specific effector molecules that suppress PTI. During the ongoing evolutionary contest between host and pathogen, plants have developed resistance (R) genes encoding receptors that can recognize and bind these effectors. This specific recognition triggers a cascade of defense reactions called effector triggered immunity (ETI)^3,4^. This incompatible interaction between pathogens and hosts limits or halts infection. The absence of an effective R gene product (defined as compatible infection) results in successful infection and colonization.

Plants protect themselves against biotic and abiotic challenges by a diverse array of defense and stress responses. These responses comprise both very rapid changes in gene expression to quickly adapt to the challenges and sustained transcriptional responses to cope with prolonged stress. Although both early and late transcriptional responses are required for optimal defense, early response genes hold the key for perceiving and amplifying the different stress signals and inducing downstream gene expression^5–7^. For example, Li et al.^6^ showed that 37R genes and many genes involved in defense signaling were induced at the early stage of infection (12 hpi). These included genes encoding *MAPKs*, genes involved in ROS/NO and hormone signaling pathways and genes associated with the synthesis of defense-related metabolites, such as phenylpropanoids/stilbenoids/flavonoids^6^. These data highlight the importance of focusing on the characterization of the early mechanisms deployed by grapevine to respond to downy mildew.

Early studies investigating the mechanisms of grapevine resistance to *P. viticola* examined histological and ultrastructural aspects, including callose deposition in stomata, lignification, stilbenic phytoalexin production, hydrogen peroxide (H_2_O_2_) accumulation, and hypersensitive reactions (HRs)^8–10^. More recent studies have utilized sequencing technologies to address molecular genetic aspects, leading to greatly enhanced understanding^5,6,11–16^. Figueiredo et al.^11^ identified differences in gene expression and metabolite profiles between resistant and susceptible grapevine cultivars using a combination of cDNA microarray and nuclear magnetic resonance spectroscopy. Wu et al.^12^ characterized gene expression in response to *P. viticola* infection in *Vitis amurensis* using Solexa sequencing technology, and showed that the differentially expressed genes were mostly associated with ribosome structure, photosynthesis, and amino acid and sugar metabolism. Similarly, to identify genes and pathways associated with downy mildew resistance, Li et al.^6^ used RNA-based sequencing (RNA-seq) to identify transcriptional responses to infection in a resistant genotype^6^.

Although transcriptional profiling studies have provided new insights into the grapevine response to downy mildew, mRNA abundance does not always reflect the expression level of the respective protein product(s). Protein levels can be influenced by various factors, including the rate of translation and stability of the protein. Proteomic techniques that encompass the extraction and purification of proteins, cleavage to peptides, and detection by mass spectrometry have been well established^17^. In recent years, proteomic-based approaches have been used to study the grapevine response to downy mildew^18–23^. Using two-dimensional gel electrophoresis (2-DE), Milli et al.^20^ identified 82 differentially expressed proteins in grapevine leaves 24, 48, and 96 h after inoculation with *P. viticola*. Xu et al. (2015) identified nine proteins expressed at different levels between a susceptible (*V. amurensis* “Shuangyou”) and resistant (*V. amurensis* “Shuanghong”) grapevine genotype after *P. viticola* inoculation using 2-DE followed by MALDI-TOF/TOF^18^. These 2-DE studies have limitations and are more suitable for the identification of abundant proteins, as they rely on the separation and staining of proteins directly in a gel. A more recent proteomic approach, designated iTRAQ (isobaric tags for relative and absolute quantitation), overcomes some limitations of 2-DE-based techniques. iTRAQ is highly sensitive and allows for the identification and quantitation of upto eight samples simultaneously^24^.

*Vitis* species and cultivars vary in resistance to *P. viticola*. The Chinese wild grapevine *V. davidii* “LiuBa-8” (LB) is highly resistant to *P. viticola*, while the *V. vinifera* cultivar “Pinot Noir” (PN) is relatively susceptible^25,26^. In the present study, we used the iTRAQ approach to quantify and assess differences in the proteomes between LB and PN at an early stage in response to *P. viticola*. We aimed to gain insight into the early molecular events and to identify candidate proteins involved incompatible and compatible interactions. These proteins could be exploited as markers to develop strategies to protect grapevines against downy mildew.

## Results

### Colonization in LB and PN at 12 hpi

**Fig. 1:**
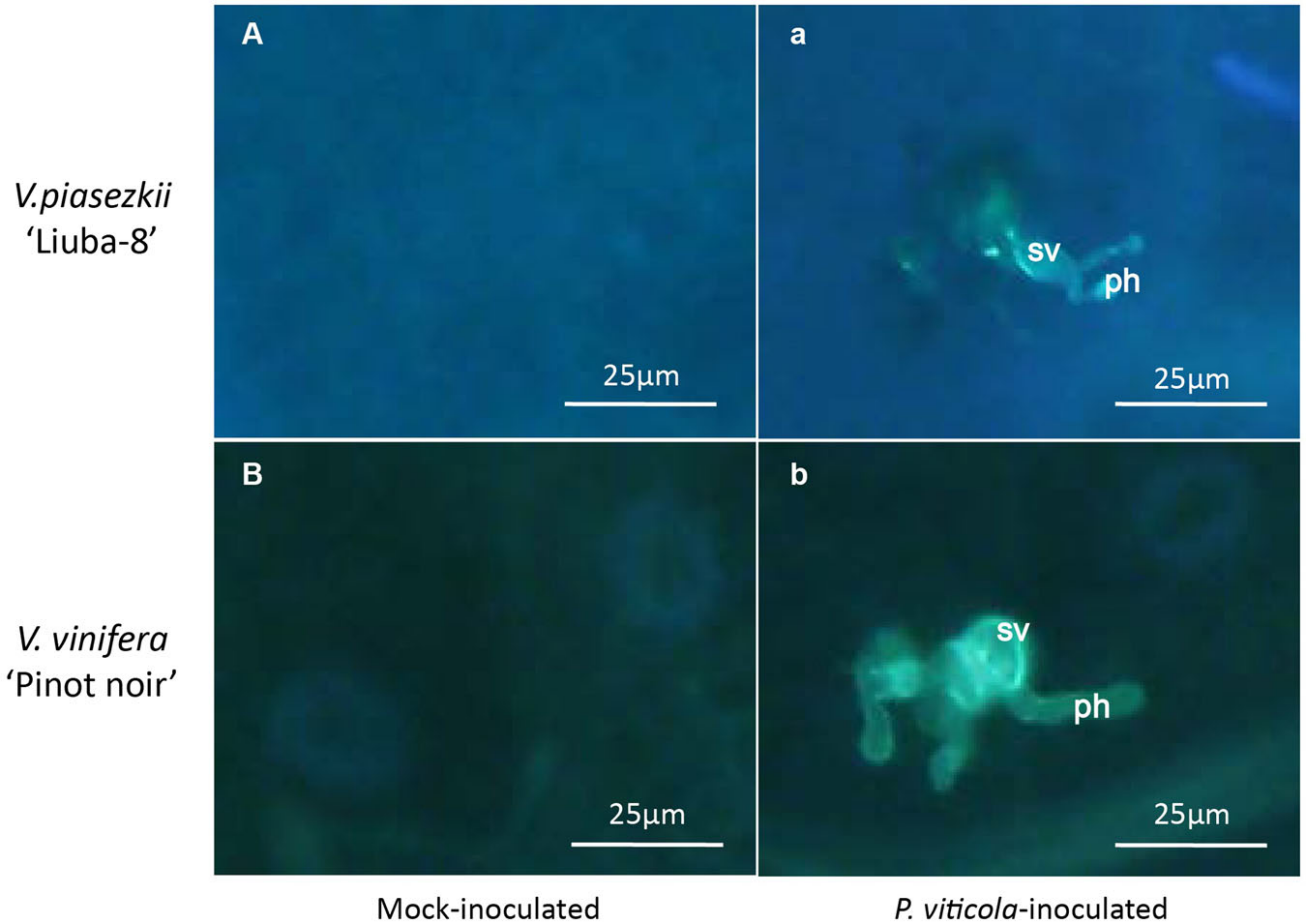
Fluorescence micrographs of leaves of *V*. *piasezkii* “Liuba-8” (A and a) and *V*. *vinifera* “Pinot Noir” (B and b) at 12 hpi. sv substomatal vesicle, ph primary hypha

To evaluate the ability of *P. viticola* to colonize LB and PN, detached leaves from both genotypes were inoculated with cultures of *P. viticola* isolate “YL”, and then observed for 12 h postinoculation (hpi) by aniline blue staining and epifluorescence microscopy. In both LB and PN, zoospores were observed near stomata, and germ tubes, primary hyphae and the first haustorium could be detected (Fig. 1). These observations are consistent with previous reports showing that *P. viticola* can successfully infect and colonize both resistant and susceptible grapevine genotypes^5,27,28^, and suggest that resistance to downy mildew in the LB-resistant genotype involves mechanisms, that are deployed after infection and colonization rather than constitutive physical and chemical barriers.

### H_2_O_2_ production in LB and PN at 12 hpi

**Fig. 2:**
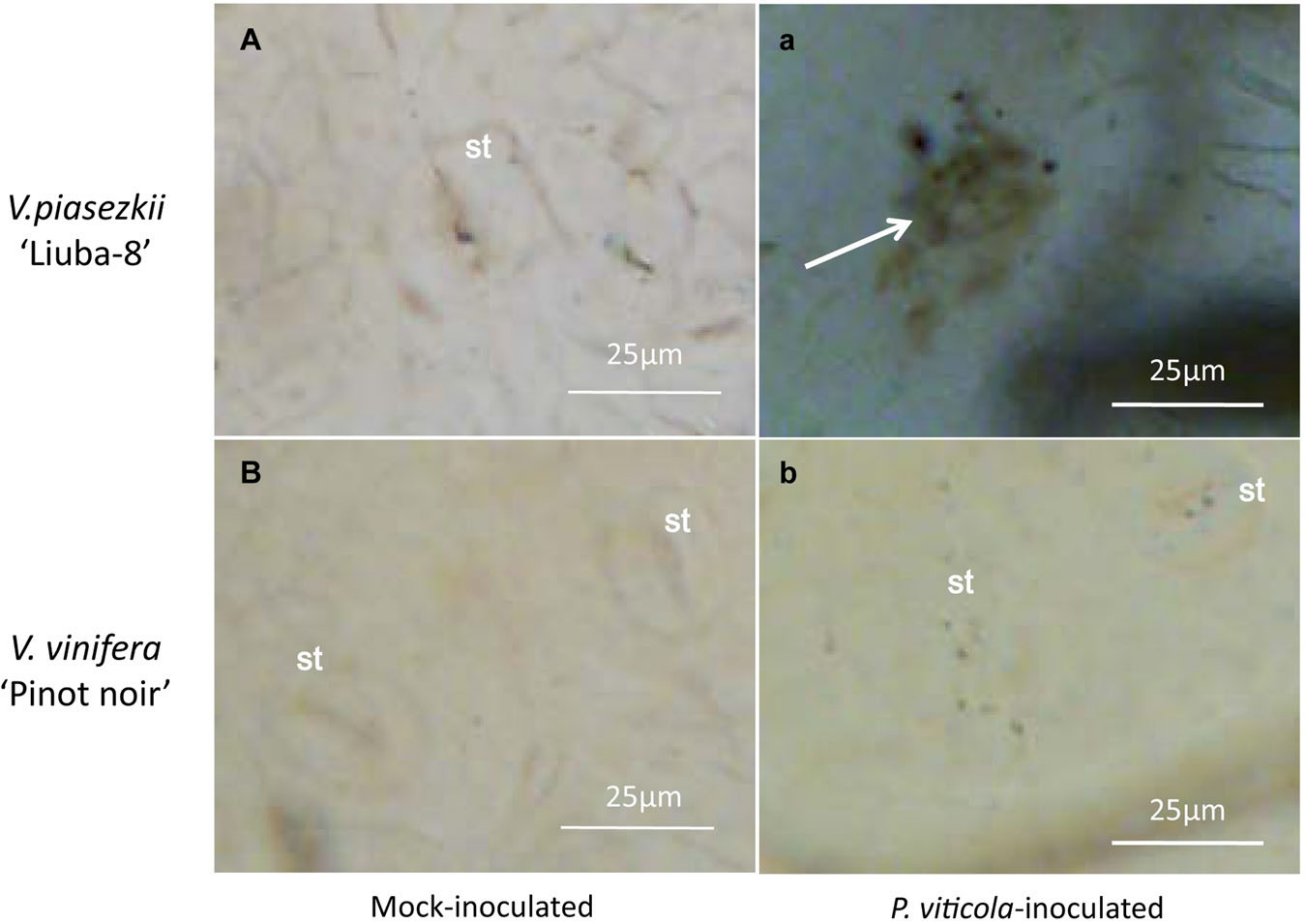
Microscopic detection of H_2_O_2_ accumulation in leaves of *V. piasezkii* “Liuba-8” (A and a) and *V. vinifera* “Pinot Noir” (B and b) at 12 hpi. st stomatum

To determine whether postinfection resistance mechanisms in LB involve the production of H_2_O_2_, leaves of LB and PN were observed 12 hpi by 3,3-diaminobenzidine (DAB) staining and light microscopy. DAB reacts with H_2_O_2_ to form an easily visible, reddish-brown precipitate. As shown in Fig. 2, reddish-brown deposits were detectable in LB leaves, while no staining was observed in PN leaves. This result revealed that *P. viticola* infection induced H_2_O_2_ production within 12 hpi in LB leaves but not in PN leaves.

### Overview of the proteomic analysis

**Fig. 3:**
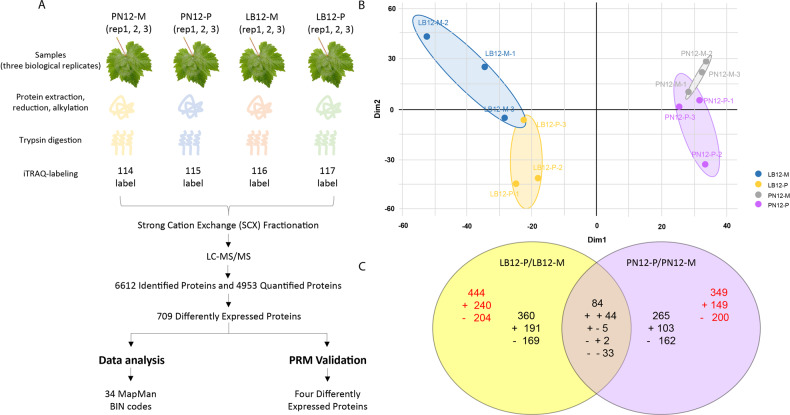
Overview of the proteomic analyses. **A** Sampling and iTRAQ workflow; **B** Principal component analysis (PCA) of the proteome datasets; **C** Venn diagrams indicating the numbers of DEPs in *V. piasezkii* “Liuba-8” and *V. vinifera* “Pinot Noir” at 12 hpi. Numbers in red indicate all DEPs for each genotype, while numbers in black indicate numbers of DEPs specific to individual genotypes

To compare the diversity and abundance of cellular proteins that accumulate during incompatible and compatible interactions between grapevine and *P. viticola* at an early stage, total proteins were extracted at 12 hpi from *P. viticola*-inoculated (P) or mock-inoculated (M) grapevine leaves (LB12-P, LB12-M, PN12-P, PN12-M, respectively) and analyzed by iTRAQ. The workflow of the analysis is shown in Fig. 3A. In total, 296,872 spectra were obtained, representing 40,165 peptides, 29,605 unique peptides and 6612 proteins. The entire dataset is accessible through ProteomeXchange (PXD018845). Principal component analysis (PCA) of the normalized protein expression data set showed that the three replicates of each experimental condition were well grouped, indicating a high degree of correlation among the replicates (Fig. 3B). Differentially expressed proteins (DEPs) were then identified based on a quantification ratio >1.2 or <0.83 at *p* value < 0.05 and with at least one unique peptide in at least two biological replicates. A total of 709 DEPs were identified in the two genotypes. Furthermore, more DEPs were observed in the resistant genotype LB (444 DEPs) than in the susceptible genotype PN (349 DEPs) (Table S1). In addition, there were more upregulated proteins than downregulated proteins in LB, while in PN, the opposite was observed. Specifically, 240 and 149 proteins were upregulated in LB and PN, respectively, while 204 and 200 proteins were downregulated in LB and PN, respectively (Fig. 3C). A total of 44 proteins were commonly upregulated in LB and PN, whereas 191 and 103 proteins were specifically upregulated in LB and PN. Among the 84 common proteins, 33 proteins were downregulated in both LB and PN, whereas 169 and 162 proteins were specifically downregulated in LB and PN, respectively. Moreover, five proteins were upregulated in LB but downregulated in PN, while two proteins were downregulated in LB but upregulated in PN (Fig. 3C).

### Functional annotation and classification

**Fig. 4:**
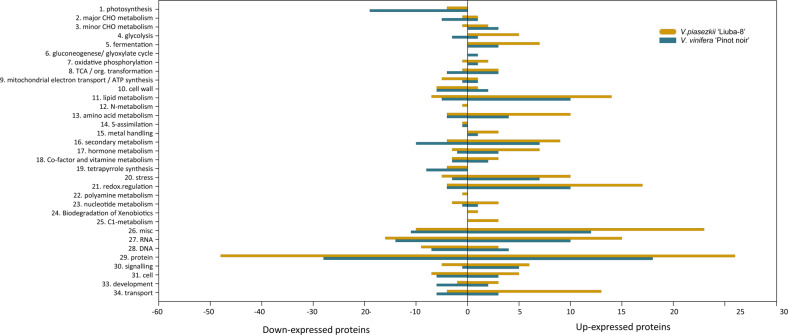
Functional categories of DEPs in *V. piasezkii* “Liuba-8” and *V. vinifera* “Pinot Noir” at 12 hpi

Among the 709 DEPs, 538 DEPs were annotated as hypothetical or unknown proteins in UniProt (http://www.uniprot.org/). To gain more information about these proteins, BLAST (http://www.ncbi.nlm.nih.gov/BLAST/) was used to identify their homologous proteins in the NCBI nonredundant (nr) protein database. In addition, to gain a more detailed description, MapMan was used to conduct the functional annotation and classification of the DEPs^29^. These 538 DEPs were classified into 34 functional MapMan bins, as shown in Fig. 4. In both LB and PN, the predominant proteins that showed alterations in abundance in response to downy mildew were assigned to photosynthesis, metabolism, stress, and redox categories.

### Validation of DEPs by parallel reaction monitoring (PRM)

**Fig. 5:**
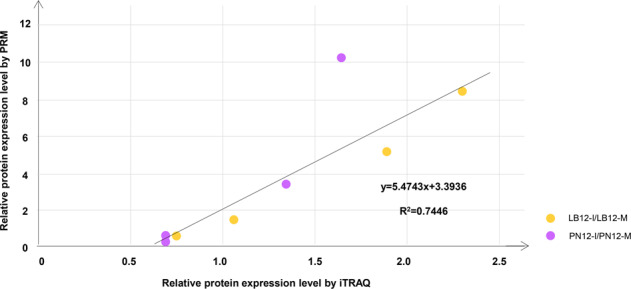
Correlation analyses between iTRAQ and PRM

PRM technology is an ion monitoring technique based on high-resolution and high-precision mass spectrometry. PRM initially uses the selective detection capability of a quadrupole mass analyzer to selectively detect the precursor ion information of the target peptide. Selected ion precursors are then fragmented by HCD in a collision cell and finally analyzed by a high-resolution and high-mass-accuracy Orbitrap analyzer. This technology allows for an accurate and specific analysis of target proteins/peptides in complex samples^30^. To validate the iTRAQ results, four differentially expressed proteins were analyzed using PRM in both varieties at 12 hpi. Data are available from ProteomeXchange (PXD018868). The correlation coefficient between the iTRAQ and PRM analyses was 0.74, which illustrates that the iTRAQ results were reliable for further analysis (Fig. 5 and Table S2).

### Transient expression of upregulated proteins confers pathogen resistance in *Nicotiana benthamiana*

To preliminarily verify the function of DEPs identified by iTRAQ, five candidate proteins were selected for transient expression in *N. benthamiana*. They were pathogenesis-related proteins (PR5, PR10.2, and PR10.3), calreticulin 2 (CRT2) and a 17.8 kDa class I heat shock protein (HSP17.8). The three PR proteins were highly induced in LB and/or PN after *P. viticola* infection and have been speculated to play pivotal roles in pathogen resistance. In addition, previous studies have shown that plant disease resistance may result from some constitutively highly expressed genes in the resistant genotype. Therefore, in our study, two candidates (CRT2 and HSP17.8), which showed constitutively high-level expression in resistant LB than PN but were hardly modulated in LB and PN in response to *P. viticola*, were also selected.

**Fig. 6:**
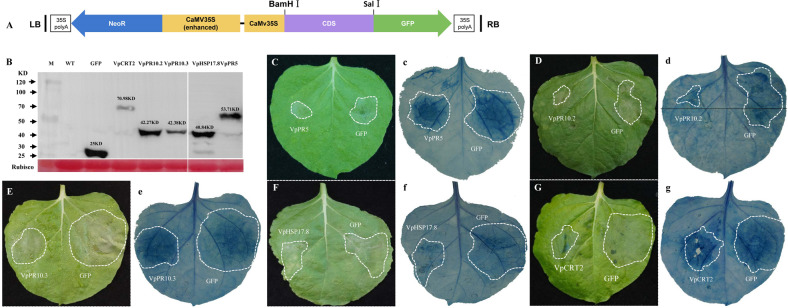
Pathogen infection analysis in transient *Nicotiana benthamiana* candidates. **A** Schematic of the plasmid used to induce transient transformation in *N. benthamiana*. **B** Western blot analysis of extracts from *N. benthamiana* leaves transiently expressing control GFP and five candidates. M marker, WT wild type. **C**–**G** Lesion development was photographed at 3–5 days after infection, and the lesion area is indicated with dotted circles. c–g Trypan blue staining of the lesions. The experiments were repeated more than three times with similar results

Genes encoding the five proteins were cloned from LB and PN first (one gene could not be cloned in PN). Sequence alignment showed that the identities of the genes in the two different genotypes reached 90% (Supplementary Fig. S1). The genes were then cloned into pCAMBIA2300, which harbors a GFP expression cassette (Fig. 6A), and transformed into *N. benthamiana* leaves by Agrobacterium-mediated plant genetic transformation. *A. tumefaciens* cells carrying only GFP were infiltrated into the right panel of the leaf as a control, while cells carrying GFP and genes were infiltrated into the left panel of the same leaf. Western blot analysis showed that all the proteins were successfully expressed in *N. benthamiana* (Fig. 6B). Zoospores of *Phytophthora capsici* were inoculated onto the agroinfiltrated leaves at 2 days of post-infiltration (dpi). The lesions showed a smaller range in the candidate-transformed leaves than in the control (Fig. 6C–G (c–g)), indicating that overexpression of these candidates in *N. benthamiana* leaves significantly impeded the growth of *P. capsici*, suggesting that these candidates may contribute to disease resistance in plants.

## Discussion

*P. viticola* is a strictly biotrophic oomycete pathogen that can only survive on living host tissues. Although there have been numerous proteomic studies of plant–pathogen interactions in recent years, few have addressed incompatible and compatible interactions at an early stage of *P. viticola* infection in grapevine, and none have employed iTRAQ. The results of our proteome analysis are discussed below and focus on the various functional groups of proteins.

### Photosynthesis inhibition was stronger in the susceptible PN than in the resistant LB

**Fig. 7:**
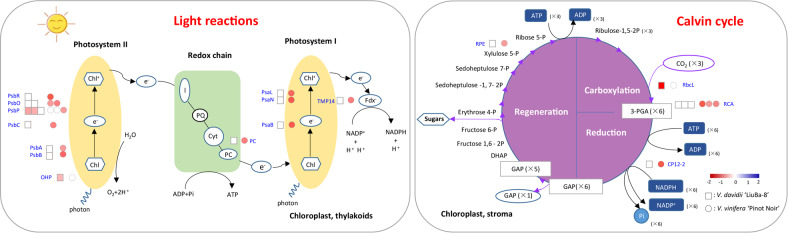
Schematic representation of DEPs involved in photosynthesis in *V. piasezkii* “Liuba-8” and *V. vinifera* “Pinot Noir” at 12 hpi

Photosynthesis is closely associated with plant productivity and energy utilization and is one of the most biotic^31^ and abiotic^32,33^ stress-sensitive physiological processes in plants. Plants are under constant attack by biotic agents such as fungi, bacteria, and viruses, and under these circumstances, the immune system responds quickly to protect against further damage. To be able to withstand this situation, plants allocate more resources from growth to defense, concomitant with a global reduction in photosynthetic capacity. A decrease in photosynthesis has been reported in both incompatible and compatible interactions^34–36^. In this study, 4 and 19 photosynthesis-related DEPs were identified in resistant LB and susceptible PN, respectively, and all of these DEPs were repressed at the early stage irrespective of compatible or incompatible infection (Fig. 7 and Table S3).

As the primary unit of photosynthesis, PSII is involved in energy absorption, conversion, and photosynthetic electron transport in the light reaction, and its activity and function can be affected by biotic and abiotic stresses. In this study, more than half of the photosynthesis-related proteins identified (3/4 in LB and 8/19 in PN) belonged to PSII, which is consistent with previous studies^20,21^. The PSII core complex comprises PsbA (D1) and PsbB (D2) and the cytochrome b559 subunits a and b. The decrease in abundance of PsbA and PsbB proteins during pathogen infection indicates a decrease in the synthesis/degradation balance of these proteins. This lowered balance in the presence of viral infection results in PSII photoinhibition^37,38^, and consequently a decrease in the reducing power available for carboxylation activity and photorespiration^39^. Kundu et al.^40^ observed a decrease in the abundance of D1 and D2 proteins in *V. mungo* plants infected with Mungbean yellow mosaic indica virus (MYMIV). Moreover, by analyzing photochemical reactions via chlorophyll a fluorescence measurement, they found that very low levels of D1 and D2 were associated with lower actual quantum efficiency of PSII in a susceptible *V. mungo* genotype. These results strongly suggest that MYMIV inhibits PSII, which might limit energy conversion by light reactions^40^. Consistent with these reports, our results showed that D1 and D2 had lower expression in susceptible PN but not in resistant LB. Oxygen-evolving complex (OEC) activity is closely associated with PsbO and PsbP. PsbO is a key structural component of many different types of OECs and functions to stabilize the manganese cluster and modulate the Ca^2+^ and Cl^−^ requirements for oxygen evolution. *N. benthamiana* plants silenced for PsbO are susceptible to several viruses^41^. In the present study, the abundance of two isoforms of PsbO was decreased in susceptible PN but was not altered in resistant LB, which suggests that PsbO is required for a basal defense mechanism in grapevine *P. viticola*, as a previous study has reported^41^. PsbP is highly conserved in higher plants and is essential for PSII core assembly and stability. Transient silencing of PsbP in *N. benthamiana* plants enhances pathogenicity and viral DNA accumulation, while overexpression of PsbP impedes disease development during the early phase of infection, suggesting that PsbP participates in the defense response during geminivirus infection^42^. However, PsbP was downregulated in thaumatin-like protein-overexpressing transgenic rice, which exhibited enhanced resistance against bacterial blight^43^. In this study, two PsbP isoforms were slightly decreased in resistant LB, and one PsbP isoform was slightly decreased in susceptible PN. Due to the different functions of PsbP in different plant-pathogen interactions, the specific role of PsbP in the grapevine response to downy mildew needs further investigation. The light-harvesting complex (LHC) is a series of proteins and chlorophyll molecules embedded in the thylakoid membrane in plants, and transfers light energy to a chlorophyll a molecule at the reaction center of the photosystem. However, in our study, only one LHC was found to be downregulated in LB and PN, which may indicate that the LHC is more stable than the PSII core complex in the grapevine response to downy mildew.

Ribulose-1,5-biphosphate carboxylase oxygenase (RubisCO) plays a crucial role in carbon dioxide fixation in the Calvin cycle. RubisCO activase (RCA), which is specifically involved in the activation and maintenance of RubisCO by carbamylation, mediates the defense response to fungi by restoring the catalytic competence of RubisCO by using energy from ATP hydrolysis^44^. It has been reported that genotypes showing a high RCA intensity may have more efficient carbon metabolism and better defenses against pathogens^45^. In contrast, a reduced RCA abundance was reported in compatible plant-virus interactions^37^. Consistent with these reports, our results also showed that the three identified RCA isoforms were all downregulated in PN, but not LB, in response to *P. viticola*.

In general, repression of photosynthesis is an active response to stress perception rather than a secondary physiological response to tissue damage. Once an attack is perceived, plant metabolism must balance potentially competing demands for resources to support defense versus cellular maintenance, growth, and reproduction. In addition, there were fewer repressed proteins in LB than in PN, which indicates that resistance may be associated with a higher recovery capacity. This can also partly explain the smaller effect on the photosynthetic rate observed for cultivars with high resistance than those with low resistance^46,47^. Our findings, together with data from the literature showing significant differences in photosynthetic protein expression between susceptible and resistant grapevine cultivars, suggest that plant responses to biotic stress are not merely physiological but instead are the results of different genetic reprogramming strategies between cultivars.

### Glycolysis, the TCA cycle, and the PPP were more induced in resistant LB than in susceptible PN

Carbohydrate metabolism plays an important role during plant interactions with pathogens. Increases in carbohydrates not only supply massive energy to defense responses, but also to regulate the expression of resistance-related genes.

Glycolysis is a network of reactions with possible sites for substrate movement in and out of various subcellular compartments. Induction of glycolysis in the cytosol facilitates plant acclimation to environmental stress^48^. It has been proposed that the regulation of glycolysis in the leaf sheaths of *R. solani*-infected rice plants is directly involved in the regulation of carbon allocation to other pathways, and that this is an important resistance response mechanism^48^. In our study, all glycolysis-related DEPs, including ATP-dependent 6-phosphofructokinase (PFK), glyceraldehyde-3-phosphate dehydrogenase (GAPDH), and pyruvate kinase (PK), were specifically induced in LB (Table 1). In contrast, only one 2–3 biphosphoglycerate-independent phosphoglycerate mutase (PGAM-i) was induced in PN, while two isoforms of PK and phosphoglucomutase (PGM) were repressed in PN. Increased expression of these enzymes may suggest a strengthened glycolysis pathway, which could lead to generation of ATP and NADPH as a response to pathogen infection. GAPDH catalyzes the reversible conversion of glyceraldehyde 3-phosphate to 1,3-bisphosphoglycerate, and thus serves to breakdown glucose to supply energy and carbon for development and abiotic stress and immune responses. Milli et al.^20^ observed the upregulation of two isoforms of GAPDH in the grapevine response to downy mildew, while Figueiredo et al.^23^ showed that GAPDH levels increased in the resistant grapevine genotype “Regent” at 6 hpi^23^. Accordingly, in our study, one isoform of GAPDH was highly expressed in LB but showed no response in PN^20,23^. However, it has also been reported that GAPDH has functions independent of glycolysis, including mediating ROS signaling^49–53^. Arabidopsis GAPDH knockouts exhibited accelerated programmed cell death (PCD), and an increased electrolyte leakage response to ETI upon inoculation with *Pseudomonas syringae*^51^. Transient overexpression of cassava cytosolic GAPDH led to decreased resistance against *Xanthomonas axonopodis* pv *manihotis*, while its silencing resulted in increased disease resistance^52^. Moreover, the silencing of cytosolic GAPDHs strengthens programmed cell death and resistance in incompatible and compatible interactions^50^. All of these observations indicate that GAPDHs act as negative regulators of plant disease resistance. Whether GAPDH plays a positive role in glycolysis or a negative role in ROS signaling in the grapevine response to downy mildew needs further investigation. PK is a key regulatory enzyme of glycolysis that catalyzes the essentially irreversible stabilization of a phosphate group from phosphoenolpyruvate (PEP) to adenosine diphosphate (ADP), producing one molecule of pyruvate and one of ATP. *Capsicum annuum* cytosolic pyruvate kinase 1 (*CaPKc1*) was induced during the incompatible interaction of hot pepper and *tobacco mosaic virus* (TMV), indicating that PK could provide pyruvate at a high concentration directly to the mitochondrion, where it might be taken up as a substrate for respiration^54^. PK accumulated at higher levels in the incompatible interaction between *V. amurensis* “Shuanghong” and *P. viticola* strains “ZJ-1-2” at 12 hpi^6^. In our study, four PK isoforms were identified in LB and PN at 12 hpi. Two of these were increased in LB, while the other two were decreased in PN. This indicates that when attacked by a pathogen, the plant requires increased ATP released from pyruvate production. The induction of PK in the defense response may be related to the increased energy demands, and therefore, the observed induction of PK in LB, but not PN, may contribute to resistance in LB against *P. viticola*.

**Table 1:** Respiration-related DEPs in *V. piasezkii* “Liuba-8” and *V. vinifera* “Pinot Noir” at 12 hpi

Accession	Protein name	Description	LB12-P/LB12-M	*p* value	PN12-P/PN12-M	*p* value
D7TBD7	PFK3	ATP-dependent 6-phosphofructokinase 3	1.3387	0.0245	1.1625	0.0467
D7SXA1	PFK5	ATP-dependent 6-phosphofructokinase	1.6847	0.0085	1.2523	0.1343
F6HG44	GAPDH	Glyceraldehyde-3-phosphate dehydrogenase	1.9687	0.0011	1.2916	0.3277
C5DB67	PGAM-i	Putative 2-3 biphosphoglycerate independant phosphoglycerate mutase	1.1062	0.2725	1.3002	0.0186
F6HDW1	PK	Pyruvate kinase	0.9113	0.2215	0.8097	0.0462
A5BTB0	PK	Pyruvate kinase	1.1932	0.0146	0.7518	0.0290
F6HVY1	PK	Pyruvate kinase	2.0420	0.0137	1.3071	0.2677
D7TIZ5	PK	Pyruvate kinase	1.2626	0.0037	0.9238	0.1013
D7T1T9	PGM	Phosphoglucomutase, chloroplastic isoform X2	1.0259	0.7138	0.8204	0.0466
D7U830	PDHA1	Pyruvate dehydrogenase E1	0.9019	0.0115	0.8121	0.0018
F6HI27	PDHA1	Pyruvate dehydrogenase E1 component subunit beta-3, chloroplastic	0.9350	0.0434	0.7605	0.0235
F6I5U2	PDHX	Dihydrolipoamide acetyltransferase component of pyruvate dehydrogenase complex	1.0167	0.8694	1.2357	0.0174
D7TEL2	ACO	Aconitate hydratase	1.5400	0.0002	1.2926	0.0234
B6VJT4	SDH3	Succinate dehydrogenase subunit 3	1.0634	0.0046	1.3335	0.0009
D7SHR6	CA	Carbonic anhydrase	1.8017	0.0003	0.9285	0.6448
D7TU30	CA	Carbonic anhydrase	0.9075	0.5078	0.8085	0.0138
D7TBH4	ME	Malic enzyme	1.4795	0.0481	0.9893	0.9609
D7U0C2	ME	Malic enzyme	0.6962	0.0332	0.6656	0.0164
D7UBH2	G6PD	Glucose-6-phosphate 1-dehydrogenase	1.2695	0.0168	1.2169	0.0188
F6HGH4	6PGD	6-phosphogluconate dehydrogenase, decarboxylating	1.3551	0.0027	1.1888	0.0350
F6HFJ9	RPI2	Ribose-5-phosphate isomerase 2	0.7215	0.0391	0.8295	0.0695
A5B1B8	HACL	2-hydroxyacyl-CoA lyase	1.4256	0.0116	1.0509	0.4342
Q43690	ADH	Alcohol dehydrogenase 1	1.3762	0.0001	1.0019	0.9872
A5C0I8	ADH	Alcohol dehydrogenase 2	1.8392	0.0008	1.3868	0.0380
Q9FZ00	ADH	Alcohol dehydrogenase 3	1.3686	0.0134	1.1509	0.2686
F6HPN2	ALDH2B4	Aldehyde dehydrogenase family 2 member B4, mitochondrial isoform X1	1.3408	0.1442	1.5281	0.0107
D7TCD6	ALDH2A1	Aldehyde dehydrogenase family 7 member A1 isoform X1	1.2873	0.0251	1.1766	0.1921
D7TJI9	PDC1	pyruvate decarboxylase 1	1.6776	0.0007	1.2187	0.0747
Q9FVE1	PDC1	Pyruvate decarboxylase 1 (Fragment)	1.8848	0.0014	1.3436	0.0487
D7TMQ2	CS	Citrate synthase	1.1094	0.0600	1.4335	0.0005
B6VJT7	Cytb	Cytochrome b	1.2250	0.0491	1.0157	0.8221
D7TFJ1	Cytbc1	Cytochrome b-c1 complex subunit Rieske, mitochondrial	0.9003	0.0126	0.8210	0.0007
F6HC12	Cox6b1	Cytochrome c oxidase subunit 6b-1 isoform X1	0.8201	0.0435	0.9918	0.9353
F6HC13	Cox6b1	Cytochrome c oxidase subunit 6b-1 isoform X3	0.7543	0.0137	0.7965	0.1050
A5ASQ0	NDUFA13B	NADH dehydrogenase [ubiquinone] 1 alpha subcomplex subunit 13-B	0.7919	0.0242	0.9631	0.4828
F6HVC5	NDUFA6	NADH dehydrogenase [ubiquinone] 1 alpha subcomplex subunit 6	0.7782	0.0476	1.0859	0.4411
A5AT60	NDUFB10B	NADH dehydrogenase [ubiquinone] 1 beta subcomplex subunit 10-B	0.6457	0.0239	1.0655	0.5699
A5BVI0	PHB	Prohibitin	0.9570	0.2767	1.2479	0.0070

The tricarboxylic acid (TCA) cycle, which is the second stage of aerobic respiration, is the major energy-producing pathway and generates most of the reduced coenzymes that will be oxidized by the electron transport chain to produce ATP. Carbonic anhydrase (CA) is an enzyme that assists the rapid interconversion of CO_2_ and H_2_O into carbonic acid, protons and bicarbonate ions. Restrepo et al. reported that silencing CA in *N. benthamiana* decreased resistance to *P. infestans*^55^, suggesting that CA is necessary for the resistance response and that pathogens may target this enzyme for suppression in compatible interactions. In our study, one isoform of CA was highly expressed in LB, while the other showed a slight decrease in PN, which supports this hypothesis. Additionally, we identified four and seven DEPs in LB and PN, respectively. In LB, three of the four DEPs were increased, while in PN, four of the seven were decreased. Earlier studies have reported that upregulation of these proteins triggers the TCA cycle to provide additional energy for the defense response through the production of pyruvate and NADPH^40,56^.

The pentose phosphate pathway (PPP) is the main route for the production of phenolic compounds to activate defense mechanisms. Two enzymes of the PPP, glucose-6-phosphate 1-dehydrogenase (G6PD) and 6-phosphogluconate dehydrogenase (6PGD), were induced in LB, while only G6PD was slightly induced in PN. This result is in agreement with previous findings showing that resistant genotypes exhibited high PPP enzyme activities^57^. G6PD and PGD play key roles in the conversion of glucose-6-phosphate to ribulose-5-phosphate, yielding NADPH for reductive biosynthesis and maintenance of the cellular redox state. Pyruvate decarboxylase (PDC) is a homotetrameric enzyme that catalyses the decarboxylation of pyruvic acid to acetaldehyde and carbon dioxide in the cytoplasm of prokaryotes and in the cytoplasm and mitochondria of eukaryotes. Overexpression of PDC in potato conferred a lesion mimic phenotype followed by activation of multiple defense responses leading to significant resistance to *P. infestans*^58^. In our study, PDC was strongly increased in LB and only marginally increased in PN. Increased expression of these enzymes suggests a strengthened PPP. The PPP furnishes NADPH to the plasma membrane NADPH oxidase to produce H_2_O_2_, which can act as a signaling molecule for disease resistance. Activation of the PPP also reflects increased demand for precursors of amino and nucleic acid synthesis in infected plants.

### Cell wall-related XTH and BXL1 may play a negative role in the grapevine response to downy mildew

Cell wall modification is an important feature of plant adaption to various environmental changes. Structural adjustments of the existing cell wall mediated by cell wall-modifying proteins allow plants to adjust to environmental changes by regulating growth and controlling the entry of biotic agents^59^. Xyloglucan plays a key role in the structure of plant cell walls by cross-linking cellulose microfibrils. Xyloglucan endotransglucosylase/hydrolase (XTH) can decompose xyloglucan chains that are not tightly bound to cellulose and assemble new xyloglucans into the cell walls and is important for the regulation of cell wall strength, extensibility, and tissue integrity^60^. Expression of DkXTH8, a persimmon XTH, in Arabidopsis resulted in increased membrane permeability^61^, while downregulation of XTH NtXET1 in tobacco resulted in the strengthening of cell walls^62^. Polygalacturonase-inhibiting proteins (PGIPs) directly limit the effective ingress of fungal pathogens by inhibiting cell wall-degrading endopolygalacturonases (ePGs). Transgenic tobacco plants expressing grapevine VvPGIP1 exhibited higher resistance to Botrytis infection, which was associated with downregulation of XTH and a decrease in XTH activity (Alexandersson et al.^63^). Related to xyloglucan modification, beta-d-xylosidase (BXL1), which is a key enzyme remodeling xylans, was also downregulated. In our study, XTH and BXL1 were highly induced in PN, while no induction was observed in LB (Table 2). These collective results suggest that XTH and BXL1 may play a negative role in the grapevine response to downy mildew. However, this hypothesis needs further investigation.

**Table 2:** Cell wall-related DEPs in *V. piasezkii* “Liuba-8” and *V. vinifera* “Pinot Noir” at 12 hpi

Accession	Protein name	Description	LB12-P/LB12-M	*p* value	PN12-P/PN12-M	*p* value
E0CR04	USPase	UDP-sugar pyrophosphorylase-like	1.2520	0.0032	0.9965	0.9784
F6I6Y4	CESA	Cellulose synthase	0.7945	0.0444	0.7553	0.0810
A5BUX9	EG	Endoglucanase	0.7444	0.0054	0.6423	0.0061
F6GW55	CSLA2	Glucomannan 4-beta-mannosyltransferase 2	0.4878	0.0261	0.4318	0.0376
F6I3Q0	FAL17	Fasciclin-like arabinogalactan protein 17	0.7666	0.0145	0.8645	0.2261
A5B7N6	FAL2	Fasciclin-like arabinogalactan protein 2	0.8441	0.0153	0.7453	0.0278
F6GTE2	UAM1	UDP-arabinopyranose mutase 1	0.9046	0.5487	0.8219	0.0218
F6H740	BXL1	Beta-D-xylosidase isoform X1	1.0381	0.7833	1.8019	0.0114
A5AZ70	PL	Pectate lyase	0.7407	0.0469	0.6178	0.0387
F6I4C9	XTH	Xyloglucan endotransglucosylase/hydrolase 2	1.1731	0.0722	1.7930	0.0070
F6HXK9	PE	Pectinesterase 3	0.7891	0.0140	0.8274	0.3513
D7TFE6	PAE	Pectin acetylesterase 8	1.1904	0.0841	0.7419	0.0448

### Protein metabolism was affected in both LB and PN in response to downy mildew

**Fig. 8:**
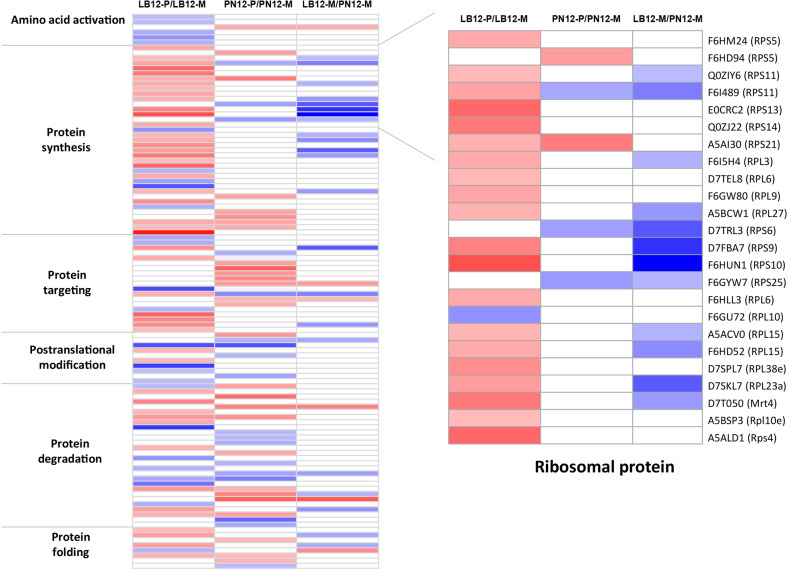
DEPs related to “protein” in *V. piasezkii* “Liuba-8” and *V. vinifera* “Pinot Noir” at 12 hpi

The MapMan bin “protein” includes amino acid activation, synthesis, targeting, posttranslational modification, degradation, folding, glycosylation, assembly, and cofactor ligation and was the most abundant category in LB (20.8%) and PN (16.7%) (Fig. 8 and Table S4). A total of 22 and 5 DEPs were characterized as ribosomal proteins (RPs) in LB and PN, respectively. Although most RPs are thought to be constitutively expressed components of core housekeeping proteins involved in translation, many studies have reported that some RPs may have functions other than ribosome structure and protein biosynthesis, playing a crucial role in the pathogen response. Overexpression of ribosomal protein L13a from eggplant (StoL13a) in *V. dahliae*-sensitive potato conferred enhanced resistance to *V. dahliae* infection, which was associated with a reduction in ROS and attenuated oxidative injury^64^. In Arabidopsis, overexpression of ribosomal protein L18 from cotton (GaRPL18) conferred enhanced resistance to *V. dahlia* infection, while silencing of GaRPL18 increased susceptibility to *V. dahliae* compared with the control by decreasing the abundance of immune-related molecules^65^. However, in our study, all RPs except RPL10 were repressed in LB (Table S4). Similar to our results, 28 of 34 RPs exhibited decreased expression in the resistant cucumber line SSL508-28^66^. The functions and mechanisms of RPs in stress responses remain largely unknown, and further studies are required to understand the role of differentially expressed RPs during the grapevine response against downy mildew. Previous studies have found that protein phosphatases 2C (PP2Cs) are also involved in plant-microbe interactions. Transgenic expression of two rice PP2Cs, OsBIPP2C1 and OsBIPP2C2, in tobacco conferred enhanced resistance to tobacco mosaic virus and *P. parasitica*^67,68^. In our study, one PP2C protein was highly induced in LB, while no induction was observed in PN, suggesting a putative role for PP2C in the disease resistance response.

### Stress-related proteins were highly expressed upon infection in both resistant LB and susceptible PN

Pathogenesis-related (PR) proteins play pivotal roles in the plant response to pathogen challenge. PR proteins are divided into 17 classes, PR1–PR17, based on their amino acid sequence, serological relationship, and enzymatic activities^69^. Four PR proteins (PR4, PR5, PR10.2, and PR10.3) were identified in our study. PR4, which is a chitinase and chitin-binding protein, plays a significant role in pathogen responses in many plants. Transgenic overexpression of PR4 in grapevine conferred increased resistance to powdery mildew, while loss-of-function PR4 mutations increased susceptibility to downy mildew in grapevine^70,71^. In our study, PR4 was induced upon infection in LB but not PN (Table 3). Moreover, even in mock-inoculated plants, PR4 was more abundant in LB than in PN. Based on this observation, we speculate that PR4 has a basal function in pathogen resistance in grapevine. PR5, also called thaumatin, is another well-characterized defensive protein in various plants. Transgenic plants expressing thaumatin at high levels exhibited enhanced resistance against various pathogens^72–74^. PR5 is supposed to produce transmembrane pores and inhibit fungal growth by restraining spore germination and germ tube growth^75^. PR5 has been widely reported to be induced in response to *P. viticola* infection in grapevine, but its specific role remains obscure, as contradictory results have been reported. One study found that *PR5* was constitutively expressed in both susceptible and resistant grapevine cultivars^10^, whereas another study reported that *PR5* was expressed at lower levels during the *P. viticola* incompatible interaction than during the compatible interaction^6^. In our study, PR5 was induced more strongly in the resistant LB than in the susceptible PN. Moreover, transient overexpression of VpPR5 significantly impeded the growth of *P. capsici*, suggesting that VpPR5 confers pathogen resistance in *N. benthamiana* (Fig. 6C, c). However, the specific function of PR5 needs further investigation in the context of the grapevine response to downy mildew. PR10 is an important protein of the plant response to fungal invasion that displays antimicrobial activities and in vitro ribonuclease (RNase) activities^76–79^. This RNase activity may protect plants during PCD at infection sites or act directly upon pathogens. In grapevine, PR10 proteins or transcripts accumulate in response to various biotic stresses, including *P. viticola* infection^5,20,80,81^. However, only a subset of PR10 gene family members are induced upon pathogen infection in grapevine. He et al. reported that the induction of three PR10 genes (*VpPR10.2*, *VvPR10.2*, and *VvPR10.3*) in response to *P. viticola* was dependent on a pyrimidine-rich cis-acting element in their promoters^79^. PR10.2 was more strongly expressed in *V. pseudoreticulata* than in *V. vinifera* in response to *P. viticola* infection, thus suggesting that it contributes to the strong downy mildew resistance of *V. pseudoreticulata*. Additionally, transgenic overexpression of VpPR10.2 in a susceptible *V. vinifera* genotype enhanced resistance to *P. viticola*^79^. In our study, both PR10.2 and PR10.3 were strongly induced in LB and PN. In addition, similar to previous research results, transgenic overexpression of VpPR10.2 and VvPR10.3 conferred pathogen resistance in *N. benthamiana* (Fig. 6D, E and d, e). These combined results suggest that VpPR10.2 and VpPR10.3 may play pivotal roles in the response of grapevine to *P. viticola*.

**Table 3:** Stress-related DEPs in *V. piasezkii* “Liuba-8” and *V. vinifera* “Pinot Noir” at 12 hpi

Accession	Protein name	Description	LB12-P/LB12-M	*p* value	PN12-P/PN12-M	*p* value
A5BS35	BSP	Basic secretory protease	1.1686	0.1613	1.3090	0.0003
A5C9R1	BI-1	Bax inhibitor 1-like	1.2685	0.0186	1.0341	0.5750
D7SI01	EDS1	Protein EDS1	1.2917	0.0346	1.2653	0.0929
A5BFQ1	LRRNT_2	LRRNT_2 domain-containing protein	0.8078	0.0231	0.8854	0.3139
D7T2C8	PR4	Endochitinase A isoform X1	2.1435	0.0144	1.0627	0.6999
A5ASS2	PR5	Thaumatin	1.8192	0.0142	1.4767	0.1142
Q9FS43	PR10.2	Pathogenesis-related protein 10.2	2.2944	0.0056	1.6451	0.0145
B7SL50	PR10.3	Pathogenesis-related protein 10.3	1.5780	0.0084	1.9446	0.0049
F6HXP8	HSP17.4	17.4 kDa class III heat shock protein	1.2575	0.0493	0.9549	0.6525
A5B2N0	HSP18.2	18.2 kDa class I heat shock protein	1.4104	0.1425	1.5049	0.0255
A5AUN6	HSP70	Heat shock 70 kDa protein 1 A/1B isoform X5	1.3001	0.1353	1.3387	0.0424
A5AGD9	HSC70	Heat shock cognate 70 kDa protein	1.2647	0.0470	1.2876	0.0274
F6GV26	HSC70.2	Heat shock cognate 70 kDa protein 2	1.6785	0.0342	1.2094	0.0549
A5AEP7	HSP90.6	Heat shock protein 90-6, mitochondrial isoform X1	1.7312	0.0257	1.3159	0.0709
F6H0H3	PMT10	Methyltransferase PMT10	0.7534	0.0434	0.7963	0.0879
F6HTC6	PMT14	Methyltransferase PMT14	1.1029	0.1431	0.8011	0.0230
F6HPC4	PMT2	Methyltransferase PMT2	0.8299	0.0196	0.9919	0.9519
F6I0C7	PMT26	Methyltransferase PMT26	0.8329	0.0279	0.7959	0.0028
A5BJD9	MLP423	MLP-like protein 423	1.1341	0.1345	0.8242	0.0355
A5BJA2	STS14	STS14 protein	0.7591	0.0490	0.9046	0.7775
D7U4I8	USP	Usp domain-containing protein	1.1801	0.1098	1.5095	0.0251

In addition to proteins in the PR family, the stress-related group of DEPs included heat shock proteins (HSPs). HSPs assist in the proper folding of newly synthesized proteins, act in innate immune responses and are essential in inducing other resistance proteins. Based on molecular mass, there are five major HSP subfamilies—HSP100, 90, 70, 60, and small HSP (sHSP)—conservatively recognized as molecular chaperones. Plants respond to pathogen invasion using two distinct innate immune responses mediated by pattern recognition receptors (PRRs) or R proteins. HSPs play an indispensable role as molecular chaperones in the quality control of plasma membrane-resident PRRs and intracellular R proteins^82^. We identified four and three *P. viticola*-induced HSPs in LB and PN, respectively. HSP70 plays a crucial role in the plant response to pathogen infection^83–87^. In our study, half of the differentially expressed HSP70s were induced in both LB and PN. Moreover, HSC70-2 was more abundant in LB than in PN, which is in agreement with a previous study showing that HSC70 is especially abundant in Regent at an early stage of infection (6 hpi)^23^. HSC70 regulates Arabidopsis immune responses^88^ and participates in both positive and negative regulation of PCD and immunity signaling^89,90^. HSP90 functions in protein complexes with a large set of cochaperones. In Arabidopsis, HSP90 interacts with Mla12 resistance (RAR1) and suppressor of the G2 allele of *skp1* (SGT1) to coordinate the RPM1 function in disease resistance^91,92^. In addition, HSP90 is essential for R gene (R3a)-mediated hypersensitivity and suppresses INF1-induced cell death activation by an RxLR effector (AVR3aKI) in the *N. benthamiana* defense against *Phytophthora infestans*^93^. In our study, the HSP90 protein Hsp90.6 was especially induced in the resistant cultivar LB. Whether Hsp90 functions as a chaperone in grapevine resistance to *P. viticola* needs further investigation. sHSPs are ATP-independent chaperones that especially interact with unfolded proteins to prevent unfolding and subsequent aggregation. sHSPs are commonly associated with abiotic stresses; however, studies have also reported that sHSPs may play critical roles in plant immunity^94–97^. One hypothesis is that chaperone activity can aid the stabilization and accumulation of R proteins. Van Ooijen et al. reported that a tomato sHSP, named RSI2, confers resistance to *Fusarium oxysporum* by interacting with the LRR domain of R protein I-2, while silencing RSI2-related sHSPs in *N. benthaminana* compromised the R protein I-2-mediated HR^97^. StHSP17.8, which was highly induced in a resistant genotype of potato against late blight infection, can interact with heat shock elements (HSEs) present in the StWRKY1 promotor region to enable the functioning of StWRKY in response to potato against *P. infestants*^96^. In our study, HSP17.8 showed no difference in LB and PN after *P. viticola* infection. However, HSP17.8 had a constitutively higher expression level in LB than in PN. Moreover, transient overexpression of VpHSP17.8 significantly impeded the growth of *P. capsici* (Fig. 6F, f). All the previous findings, together with the results provided in the present study, suggest that VvHSP17.8 is required for pathogen resistance.

Calreticulin (CRT) is a highly conserved calcium-binding molecular chaperone that facilitates the folding of newly synthesized glycoproteins and regulates Ca^2+^ homeostasis in the endoplasmic reticulum (ER) lumen^98,99^. It has been reported that CRT isoforms (CRT1, CRT2, and CRT3) are important regulators of plant innate immunity. CRT2 appears to have a dual regulatory role in plant defense against biotrophic pathogens^100^. Although overexpression of CRT2 induced SA accumulation and activated some systemic acquired resistance-associated marker genes, it also resulted in increased susceptibility to *Pst* DC3000. However, in our study, CRT2 showed a higher constitutive expression level in LB than in PN. In addition, in contrast to the aforementioned results, transient overexpression of VpCRT2 increased resistance to *P. capsici* in *N. benthamiana* (Fig. 6G, g). Since there are still relatively few studies of CRT2, future studies will be required to elucidate the role of CRT2 in plant immunity.

### Fine-tuning of the redox status in the grapevine response to downy mildew

When experiencing pathogen invasion, plants deploy various defense mechanisms, including the oxidative burst leading to a rapid production of ROS (especially H_2_O_2_). The accumulation of ROS marks the successful recognition of infection and the activation of plant defense responses. ROS play an essential role in pathogen resistance by directly reinforcing cell walls through cross-linking of glycoproteins and lipid peroxidation^101^. Previous studies have shown that H_2_O_2_ accumulates more rapidly in resistant genotypes than in susceptible genotypes in many plant–pathogen interactions^23,102,103^. In grapevine, H_2_O_2_ accumulated more quickly and to higher levels in “Regent” than in a susceptible genotype^23^. H_2_O_2_ production is one of the earliest (12 hpi) detectable cytological events against downy mildew in the resistant grapevine clutivar “Solaris”^104^. In the present study, H_2_O_2_ accumulated in LB but not in PN by 12 hpi (Fig. 2). These results showed that in LB, H_2_O_2_ plays an important role in early defense against downy mildew. In general, ROS bursts constitute some of the earliest plant responses to pathogen invasion, and as signaling molecules, ROS can regulate PCD during pathogen infection. ROS levels depend on the balance between ROS production and scavenging^105^, and when excess ROS are produced, injuries will occur. ROS amounts depend both on enzymatic and nonenzymatic scavenging molecules such as superoxide dismutase (SOD), ascorbate peroxidase (APX), catalase (CAT), peroxidase (POX), and the antioxidants ascorbate (ASC), glutathione (GSH), and glutathione peroxidases (GPX), which offer a highly efficient system for maintaining ROS homeostasis^105,106^. Antioxidant enzymes are induced against pathogens in both resistant and susceptible genotypes, although at varying levels. In this study, we identified 21 and 14 differentially expressed ROS-associated proteins in LB and PN, respectively, and most of them (17 in LB and 10 in PN) showed higher expression in inoculated leaves than in the corresponding mock control (Table 4). APX is a key enzyme in the ascorbate-glutathione cycle, an important antioxidant system that is able to detoxify ROS in plant cells. In grapevine, APX has been shown to be induced by pathogen infection^20,107–109^. In our analysis, the higher expression of two APXs in LB relative to PN may contribute to resistance in LB. POX catalyzes the reduction of H_2_O_2_ and a variety of organic and inorganic hydrogen donors. During this process, substrates are oxidized, promoting lignin formation in the cell wall. Many studies have reported the involvement of POXs in pathogen responses^110–112^. These POXs may also promote changes in plant cell walls to form a physical barrier blocking invasion. We found that in PN, four of the seven identified POXs were upregulated, consistent with a role in resistance. Furthermore, we did not identify any upregulated POX in LB, suggesting that genotype-specific resistance in PN may be due at least partly to a strengthened cell wall. Glutathione S-transferases (GSTs) are an isozyme family catalyzing the conjugation of the reduced form of glutathione to a number of electrophilic substrates, such as phytotoxic compounds, for detoxification. GSTs detoxify metabolites or phytotoxins produced during oxidative damage or pathogen infection^113,114^. Previous studies have shown that GSTs contribute to resistance against powdery mildew. In wheat, *GSTF5* was more highly expressed during an incompatible interaction than during a compatible interaction^115^. Moreover, in powdery mildew-infected tomato, a GST gene was more rapidly upregulated in a resistant wild genotype harboring the *Ol-1* resistance gene than in a susceptible genotype. Virus-induced gene silencing was used to reduce the expression of this *GST* gene in resistant plants, and the *GST*-silenced plants showed a susceptible phenotype after inoculation with *O. neolycopersici*^116^. In our study, a total of 11 isoforms of GSTs were identified, and all of these isoforms showed increased expression in both resistant LB and susceptible PN. However, more GST isoforms were detected in resistant LB than in susceptible PN. These results imply that GSTs may be involved in grapevine resistance to downy mildew.

**Table 4:** Redox-related DEPs in *V. piasezkii* “Liuba-8” and *V. vinifera* “Pinot Noir” at 12 hpi

Accession	Protein name	Description	LB12-P/LB12-M	*p* value	PN12-P/PN12-M	*p* value
D7SKR5	APX2	l-ascorbate peroxidase 2, cytosolic	1.2619	0.0046	1.1535	0.0765
F6I106	APX3	l-ascorbate peroxidase 3, peroxisomal	1.2350	0.0172	1.0673	0.6705
A5AL13	GME	GDP-mannose 3,5-epimerase 2 isoform X1	1.2291	0.0406	1.0084	0.8876
A5JPK5	GME	GDP-mannose-3′,5′-epimerase	1.6526	0.0209	1.0241	0.8189
D7TS92	GR	Glutathione reductase	1.3918	0.0136	1.2166	0.0347
F6HLE2	OXP1	5-Oxoprolinase	1.2065	0.0051	1.1469	0.0234
F6HQJ3	HB2	Hemoglobin-2	3.1828	0.0465	1.9652	0.1981
D7U252	GST	Glutathione S-transferase	1.0140	0.9072	1.3040	0.0286
F6HZU2	GST	Glutathione S-transferase	1.5318	0.0968	1.9754	0.0030
D7T7H2	GST	Glutathione S-transferase	1.8345	0.0154	1.3628	0.1398
D7TQY1	GST	Glutathione S-transferase	1.5083	0.0186	1.0644	0.5396
A5AG54	GST	Glutathione S-transferase	1.3174	0.0347	1.3280	0.0878
A5BCR8	GST	Glutathione S-transferase	1.4124	0.0137	1.2549	0.0681
D7TQB6	GSTL3	Glutathione S-transferase L3	1.6252	0.0296	1.3937	0.1597
D7TQB0	GSTL3	Glutathione S-transferase L3	1.3519	0.0339	1.2717	0.0556
F6H8J1	GST	Glutathione transferase GST 23	1.8629	0.0048	1.2489	0.5001
F6HR78	GST	Gutathione S-transferase	1.2371	0.0018	1.2420	0.0411
A5AQA0	GST	Gutathione S-transferase U7	1.1900	0.0182	1.3175	0.0058
A5BPH1	GHR	Glutathionyl-hydroquinone reductase YqjG	1.3409	0.0125	1.2175	0.0009
F6HIK4	POX	Peroxidase	0.7664	0.0006	0.9086	0.2290
A5C5U0	POX	Peroxidase	0.8881	0.3360	0.7934	0.0155
F6GXY7	POX	Peroxidase	1.0961	0.3932	1.4876	0.0231
A5B8V0	POX	Peroxidase	1.6515	0.0739	1.4642	0.0466
F6GY60	POX	Peroxidase 12	0.9447	0.6975	1.3850	0.0412
A7NY33	POX	Peroxidase 4	1.0229	0.8431	1.2716	0.0262
F6GWS4	POX	peroxidase 72	0.7085	0.0389	0.8475	0.5531
G1JT87	Prx II	Peroxiredoxin	0.8595	0.2273	0.7557	0.0436
A5BAW6	TRX	Peroxiredoxin-2E, chloroplastic	0.9841	0.8486	0.6625	0.0120
D7TES2	SOD	superoxide dismutase [Fe] 3, chloroplastic isoform	0.6570	0.0165	0.6750	0.0708
E2GMW1	TRX-H	Thioredoxin h	1.1379	0.5355	0.7204	0.0061
A9UFY2	TRX-H	Thioredoxin h	1.2989	0.0232	1.1893	0.2921
D7T9N8	TO1	Thioredoxin O2, mitochondrial	0.8273	0.0331	1.1203	0.0380

## Conclusion

**Fig. 9:**
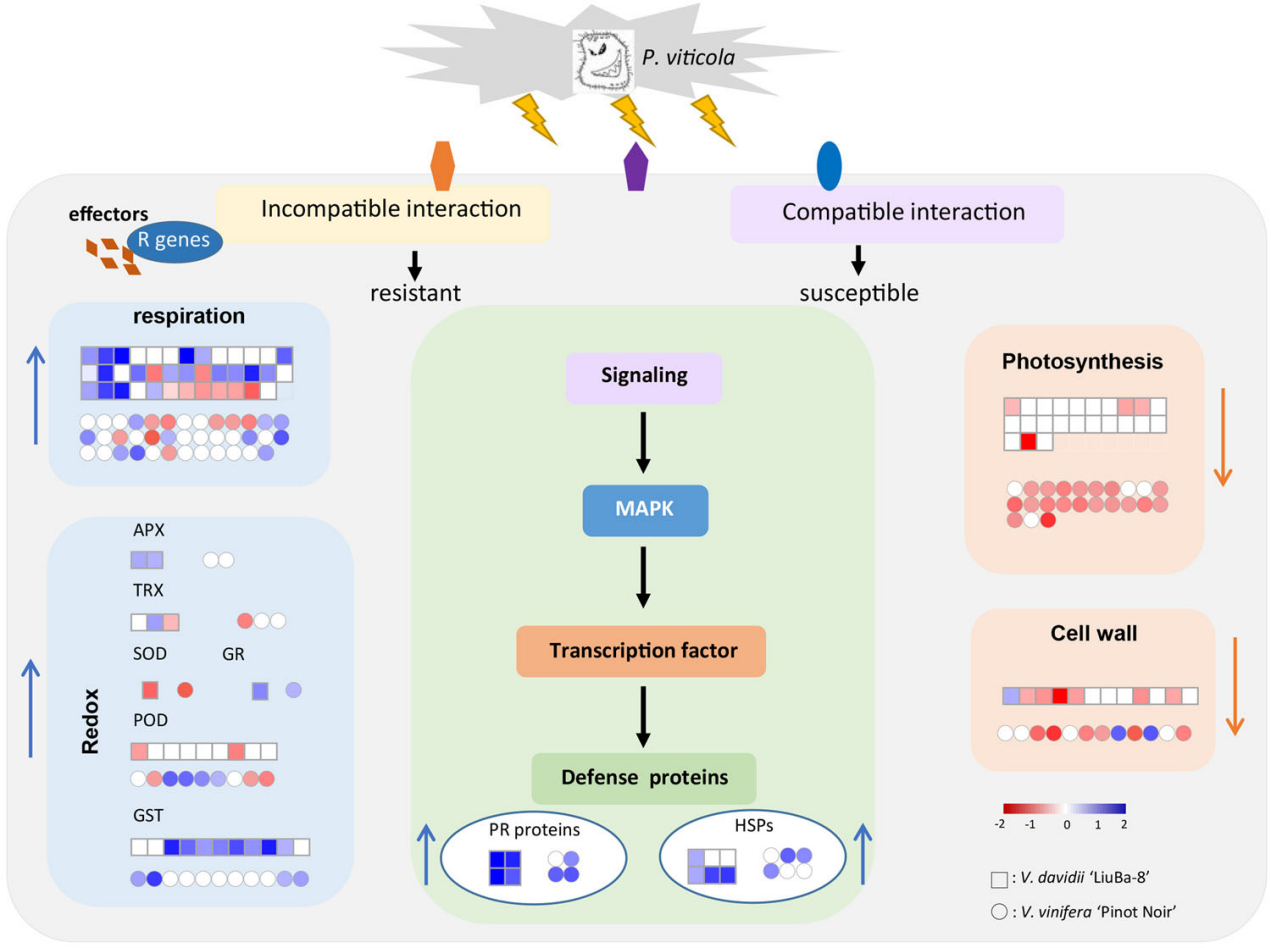
Schematic overview of DEPs in *V. piasezkii* “Liuba-8” and *V. vinifera* “Pinot Noir” at 12 hpi

Grapevine downy mildew is one of the most devastating grapevine oomycete diseases worldwide. *Vitis vinifera* cultivars are generally susceptible to the downy mildew pathogen *P. viticola*, whereas wild grapevines can show strong resistance. Comparative proteomics of grapevine leaves from the resistant genotype *V. davidii* “LiuBa-8” (LB) and the susceptible genotype *V. vinifera* “Pinot Noir” (PN) at 12 hpi were conducted to understand the complex relationship of incompatible and compatible interactions between grapevine and *P. viticola* at the early stage of infection. A total of 444 and 349 DEPs were identified in LB and PN, respectively, at 12 hpi by iTRAQ. The majority of these DEPs were related to photosynthesis, respiration, cell wall modification, protein metabolism, stress, and redox homeostasis (Fig. 9). Our broad comparative characterization of resistant and susceptible genotypes provides insights into the molecular events and identifies candidate proteins underlying incompatible and compatible interactions; these resources might be exploited to develop new protection strategies against downy mildew in grapevine.

## Materials and methods

### *P. viticola* isolates, plant materials, and sample collection

*P. viticola* isolate “YL” was selected for its consistent and high production of sporangia. It was originally isolated as a single sporangiophore from a leaf of hybrid grapevine at the Grape Repository of Northwest A&F University, Yangling, Shaanxi, China, showing typical symptoms of downy mildew according to our previous method^26^. Briefly, *P. viticola* was serially infected three times by transferring just one sporangiophore from the contaminated leaves. Next, the isolate was reproduced weekly on the separated PN leaves, which were placed in a 90 mm Petri dish (off-axis surface facing up) on wet filter paper. In addition, the isolate was maintained in a controlled environment with 80% relative humidity that was bright at 22 °C for 16 h and then dark at 18 °C for 8 h. The third and fourth leaves from the apex of the PN and LB vertices were collected. The surfaces of these leaves were disinfected with 0.01% bleach, and sterile distilled water (SDW) was then used to rinse the leaves three times. Leaf disks with a diameter of 10 mm were obtained by using a sanitized cork borer. Leaf abaxial surfaces were inoculated with 50 µL drops of an aqueous suspension of 5 × 10^4^ sporangia per mL and placed on wet filter paper in 90 mm Petri dishes. The control groups of leaf disks were inoculated with 50 µL drops of sterile distilled water. Incubation conditions were as described above. Three independent biological replicates were collected for each condition (*P. viticola*-inoculated and mock-inoculated), each comprising a pool of three leaves from three different plants.

### Visualization of *P. viticola* and localization of H_2_O_2_ in *P. viticola*-inoculated and mock-inoculated leaves

The inoculated leaves were cut into small pieces approximately 1 cm^2^ in area and were then immersed in a solution containing 1 mg/mL DAB dissolved in HCl-acidified (pH 3.8) distilled water. Leaves were incubated for 8 h to absorb DAB and react with H_2_O_2_ and peroxidase. Disks with a diameter of 10 mm were removed from the inoculated leaf centers, and the disks were fixed and decolorized in a solution of ethanol/chloroform (3:1, v/v) containing 0.15% (w/v) trichloroacetic acid for 3–5 days. Next, samples of the leaf disks were clarified in saturated chloral hydrate until they became semitransparent, and then they were placed in 0.05% aniline blue (0.1 M phosphate buffer, pH 8.0) and incubated at room temperature overnight. The disks were then mounted on microscope slides in the staining solution, with their abaxial surfaces facing upwards. For *P. viticola*, leaf discs were observed under blue-violet light by using fluorescence microscopy. The microscope was an Olympus BX-51, with an excitation wavelength of 400–440 nm and an emission wavelength of 475 nm. H_2_O_2_ production was determined by visualization of the samples under bright field conditions and indicated by a reddish-brown coloration.

### Sample preparation for iTRAQ

Total proteins were extracted using the cold-acetone method^17^. Briefly, five volumes of TCA/acetone (1:9) were added to frozen powder of the sample and then mixed vigorously. The mixture was held at −20 °C for 4 h and then centrifuged at 6000 × *g* for 40 min at 4 °C. The precipitate was washed with −20 °C cold acetone three times and centrifuged at 6000 × *g* for 40 min at 4 °C each time. The solid substance was then air dried, 25 mg of powder was combined with 750 µL of SDT buffer, and the mixture was heated to boiling for 5 min. The lysate was then sonicated and heated to boiling for 15 min. The clear liquid on the surface was filtered with a pore size of 0.22 µm after centrifugation at 14,000 × *g* for 40 min, and the protein was quantitated using a BCA Protein Assay Kit. The samples were kept at −80 °C. For each sample, a medium amount of protein (20 mg) was incorporated into 5× SDS-PAGE loading buffer, and the mixture was heated to boiling for 5 min. Then, on 12.5% SDS-PAGE gels, the proteins were separated for 90 minutes by using an invariable current of 14 mA. Proteins were visualized by Coomassie Blue R-250 staining.

### Filter-aided sample preparation (FASP digestion) and iTRAQ labeling

Approximately 200 µg of protein from each sample was incorporated into 30 μL SDT buffer (4% SDS, 100 mM DTT, 150 mM Tris-HCl pH 8.0)^117^. UA buffer (8 M urea, 150 mM Tris-HCl pH 8.0) was employed to separate DTT, detergent and other LMW elements through repeated ultrafiltration (Microcon units, 10-Kd mass cutoff). Next, to prevent the cysteine residue from being reduced, 100 μL of iodoacetamide (100 mM IAA in UA buffer) was incorporated into samples, which were kept in the dark for 30 min. One hundred microliters of UA buffer was used to clean the filters three times, and then 100 μL of DS buffer was used twice. Finally, the protein suspensions were digested with 4 μg trypsin (Promega) in 40 μL DS buffer overnight at 37 °C, and the resulting peptides were collected as a filtrate. Peptides from each sample were desalted on C18 cartridges, concentrated by vacuum centrifugation, and reconstituted in 40 Ul 0.1% (v/v) formic acid. The peptide content was estimated by UV light spectral density at 280 nm using an extinction coefficient of 1.1 and 0.1% (g/L) solution, which was calculated on the basis of the frequency of tryptophan and tyrosine in vertebrate proteins. The peptide mixture (100 μg) from each sample was labeled using iTRAQ reagent according to the manufacturer’s instructions.

### Peptide fractionation with strong cation exchange (SCX) chromatography

iTRAQ-labeled peptides were fractionated by SCX chromatography using the AKTA Purifier system (GE Healthcare). The dried peptide mixture was reconstituted and acidified with Buffer A (10 mM KH2PO4 in 25% ACN, pH 3.0) and loaded onto a PolySULFOETHYL 4.6 × 100 mm column. The peptides were sequentially cleaned using different Buffer B (500 mM KCl, 10 mM KH_2_PO_4_ in 25% ACN, pH 3.0) with a current velocity of 1 milliliter per minute. First, 0–8% Buffer B was used for the first 22 min, then 8–52% Buffer B was used from 22 to 47 min, 52–100% Buffer B from 47 to 50 min, and 100% Buffer B from 50 to 58 min, and finally Buffer B was retuned to 0% after 58 min. The elution process was supervised by measuring the absorbance at 214 nm, and fractions were generated every minute. C18 cartridges were used to desalt the generated fractions, and then vacuum centrifugation was employed to condense them.

### Mass spectrometry (MS)

The peptide combination was loaded onto a reversed-phase trap column connected to a C18 reversed-phase analytical column in Buffer A (0.1% formic acid) and separated with a linear gradient of Buffer B (84% acetonitrile and 0.1% formic acid) at a flow rate of 300 nL/min controlled by IntelliFlow technology. LC-MS/MS analysis was carried out through a Q Exactive mass spectrometer (Thermo Scientific), which functioned in positive ion pattern and was connected to an Easy nLC for 120 min. MS data were generated by adopting a data-dependent top ten approach, which conducts higher energy dissociation fragmentation by picking the rich precursor ions out from the survey scan (300–1800 *m/z*). The target automatic gain control (AGC) was adjusted to 3e6, the maximum injection duration was 10 ms, and the dynamic exclusion period was 40 s. At *m/z* 200 and at a resolving power of 70,000, survey scans were obtained. The resolving power of the higher energy dissociation spectra was configured to be 17,500 at *m/z* 200, the normalized energy of collision to be 30 eV, the width of the isolation to be 2 m/z, and the underfill ratio, which is defined as the target value’s minimum percentage that may be achieved during the maximum filling period, to be 0.1%. The equipment was operated in activated peptide recognition mode. The MS data have been deposited in the ProteomeXchange Consortium via the PRIDE partner repository with the dataset identifier PXD018845^118^.

### Data analysis

MASCOT 2.2 was implanted into Proteome Discoverer 1.4 against UniProt *Vitis vinifera* (20180122, 54701 proteins), and the decoy database was employed to search the MS/MS spectra. The following settings were used for all parameters: trypsin was the digestion enzyme; the maximum missed cleavage was 2; fixed modification: carbamidomethyl (C), iTRAQ 4/8plex (N-term), iTRAQ 4/8plex (K); variable modification: oxidation (M), iTRAQ 4/8plex (Y); peptide mass tolerance was ±20 ppm; fragment mass tolerance was 0.1 Da; and the peptide FDR was 0.01.

### Parallel reaction monitoring (PRM) analysis

To verify the protein expression levels obtained by iTRAQ analysis, four proteins with differential expression levels were further quantified by LC-PRMMS analysis^119^. Briefly, peptides were prepared according to the iTRAQ protocol, and an AQUA stable isotope peptide was spiked in each sample as an internal standard reference. Tryptic peptides were loaded on C18 stagetips for desalting prior to reversed-phase chromatography on an Easy nLC-1200 system (Thermo Scientific). Liquid chromatography gradients were applied over 1 h with acetonitrile ranging from 5 to 35% over 45 min. PRM analysis was conducted through a Q Exactive Plus MS. To employ unique peptides with great confidence and intensity for each targeted protein, an improved collision energy, retention time and charge state for the most notably regulated peptides was obtained through experiments. The MS was performed with positive ion mode, and the parameters were as follows: A complete MS1 scan was achieved at 70,000 resolving power (at 200 *m/z*), the target ACG was 3.0 × 10^−6^, and the maximum ion injection duration was 250 ms. After the complete MS scans, 20 parallel reaction monitoring scans with 35,000 resolution (at 200 *m/z*), AGC value of 3.0 × 10^−6^ and maximum injection duration of 200 ms were performed. The target peptides were then separated using a 2 Th window. In an HCD collision cell, ions were activated or dissociated at a normalized collision energy of 27. The Skyline method (MacCoss Lab, University of Washington) was used to analyze the raw data^120^. In this analysis, the signal intensities of each greatly changed protein’s single peptide sequences were quantified and normalized to a standard reference. The MS proteomics data have been submitted to the ProteomeXchange Consortium through the PRIDE partner repository^118^ with the dataset identifier PXD018868.

### Vector construction and transient transformation in *N. benthamiana*

Vectors were constructed using the ClonExpress II One Step Cloning Kit (Vazyme Biotech Co., Ltd.) according to the manufacturer’s instructions. First, the full-length CDS without the termination codon was amplified from LB and PN cDNA using high-fidelity DNA polymerase (KOD-plus, TOYOBO) and gene-specific primers (Table S5). The primers contained a pair of homologous arms and BamH1 and Sal1 cutting sites at the end of the forward and reverse homologous arms, respectively. The binary vector pCAMBIA2300, which harbors a GFP expression cassette, was digested by the restriction enzymes BamH1 and Sal1. Then, the purified CDS fragments were ligated to the digested vectors by seamless cloning. Finally, the constructed plasmids were introduced into *A. tumefaciens* GV3101 cells by the freeze-thawing method.

*N. benthamiana* plants were grown in a greenhouse with a 16-h day at 22 °C and an 8-h night at 18 °C. Leaves from 4-to-5-week-old plants were used for transient transformation by agroinfiltration. *A. tumefaciens* GV3101 carrying binary vectors was cultured in Luria-Bertani (LB) medium with kanamycin at 28 °C and 180 rpm for 18–24 h. Concentrated Agrobacterium cells were resuspended in infiltration buffer (10 mM MgCl_2_, 500 mM MES, 200 μM acetosyringone) and adjusted to a final OD600 of 0.4–0.6 before infiltration. The suspensions were infiltrated into *N. benthamiana* leaves using a syringe.

### Protein extraction and Western blotting

Agroinfiltrated *N. benthamiana* leaves were harvested at 2 dpi and ground in liquid nitrogen. Total proteins were extracted with PPEB extraction buffer according to our previous study^71^. The extracted proteins were separated using sodium dodecyl sulfate polyacrylamide gel electrophoresis (SDS-PAGE) and transferred to a polyvinylidene fluoride (PVDF) membrane. Mouse monoclonal anti-GFP antibody (Transgen Biotech) diluted at a proportion of 1:4000 in TBST buffer was incubated with PVDF membranes overnight at 4 °C and washed three times with TBST. Then, goat anti-mouse lgG (H&L)-HRP-conjugated antibody (Jiamay Biotech) was added at a ratio of 1:5000 for 2 h at room temperature. The PVDF membranes were visualized using an HRP-ECL system to verify whether the protein was expressed.

### Pathogen infection assay

*P. capsici* strains were routinely grown in V8 agar medium at 28 °C in the dark. Cultures were maintained in solid-liquid V8 medium for 7 days before zoospores were harvested and used for infection; zoospores were induced by rinsing cultures with sterile water followed by treatment at 4 °C for 0.5–1 h. Then, 30 μL droplets of zoospores were inoculated onto the abaxial side of detached leaves 48 h post-agroinfiltration, and leaves were incubated for 3–5 days on wet paper towels at 100% relative humidity. Infections were analyzed by photography. The lesioned *N. benthamiana* leaves were treated in boiling trypan blue solution (10 mL lactic acid, 10 mL glycerol, 10 mL ddH_2_O, 10 g phenol, 60 mL absolute ethyl alcohol, and 67 mg trypan blue) for 5 min. Samples were soaked in 2.5 g/mL chloral hydrate solution to decolorize and clear the background to observe the area of cell death, which was then recorded.

## Supplementary information

Supplementary Figure

Supplementary table
