# Supplementary figures and images for: Proteomic analysis of early-stage incompatible and compatible interactions between grapevine and P. viticola
Source: Hortic Res. 2021 May 1;8:100. doi: 10.1038/s41438-021-00533-y (PMC8087781; doi:10.1038/s41438-021-00533-y)

Figure S1 Sequence alignment of four DEPs genes from LB and PN


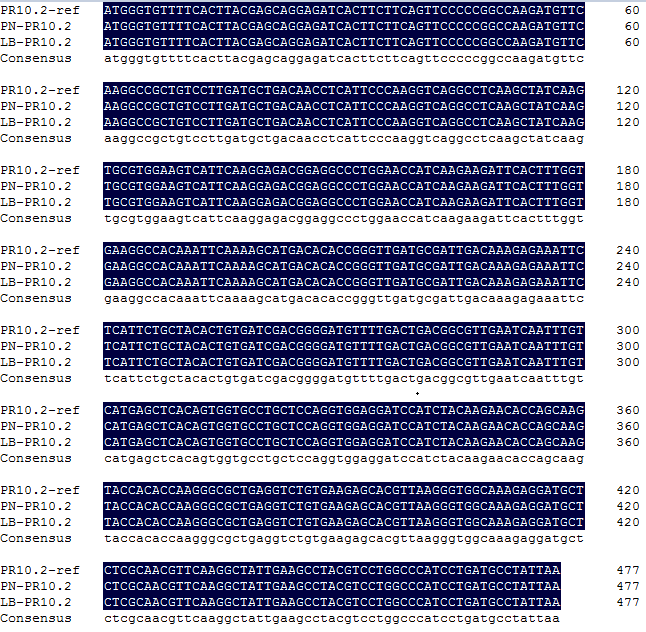


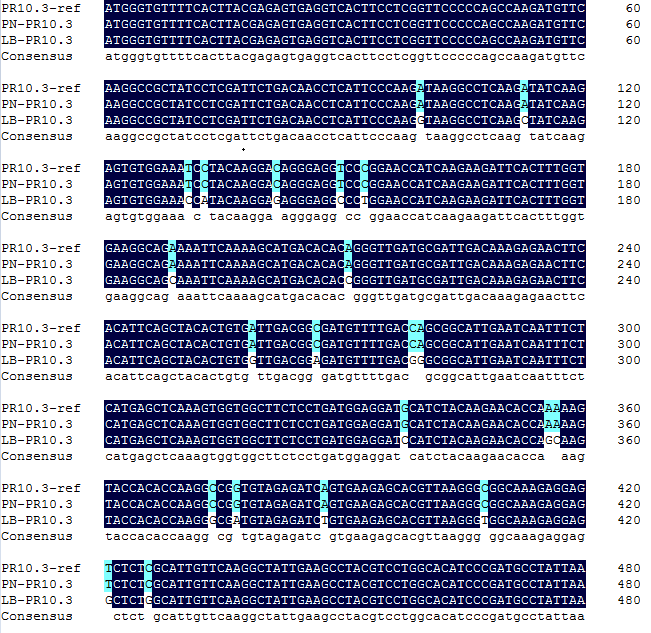


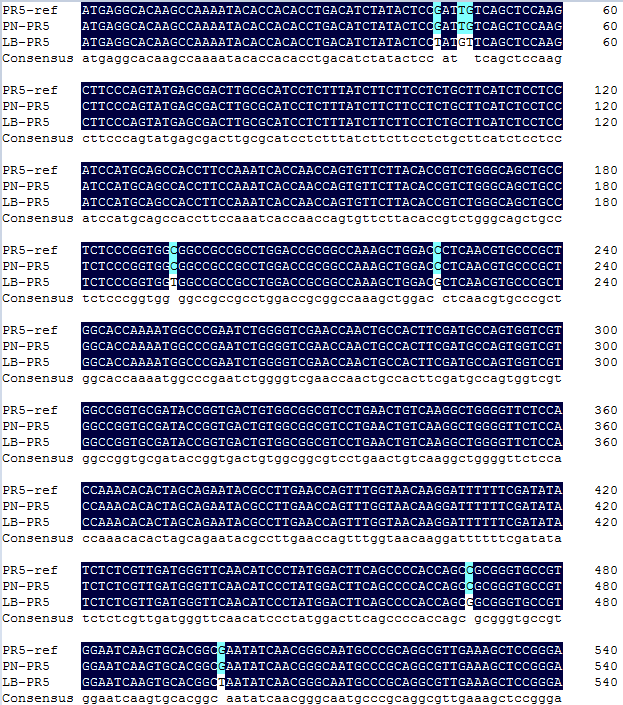

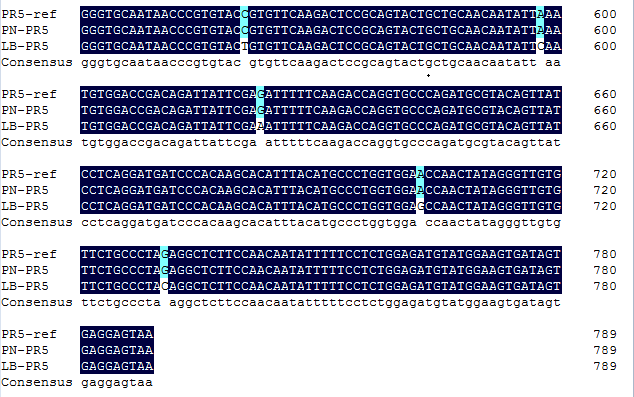


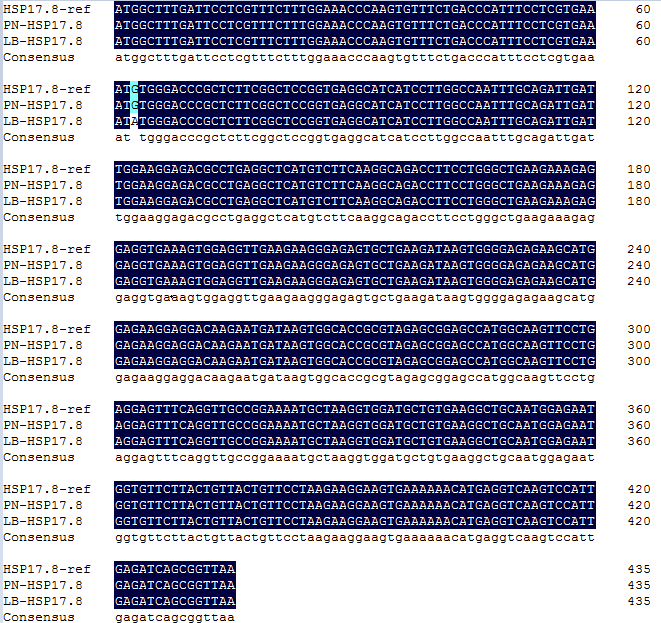


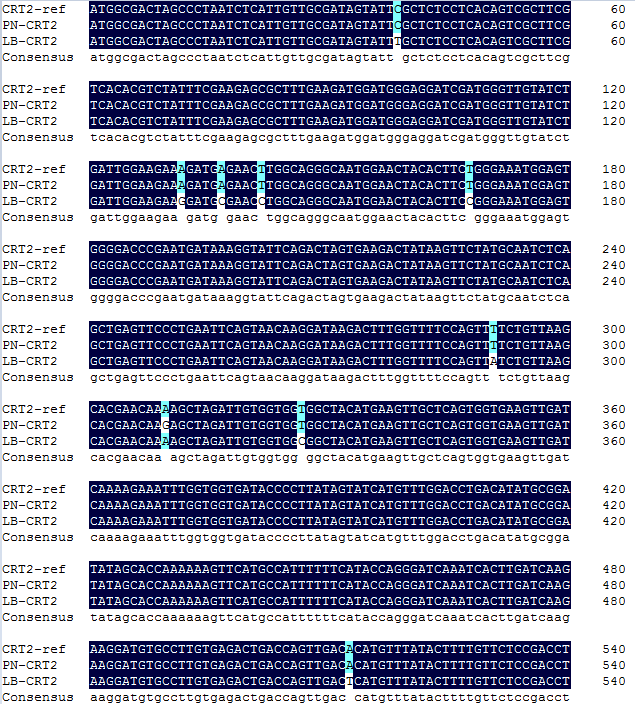

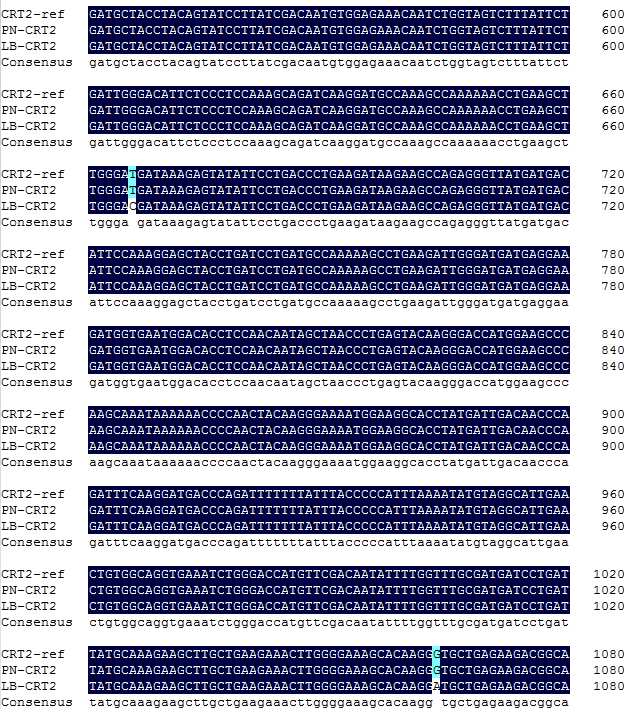

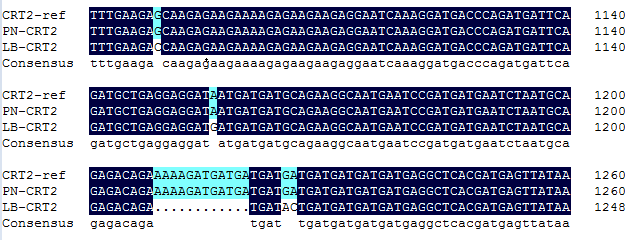

Supplement: Supplementary file 1 — Supplementary Figure [file 41438_2021_533_MOESM1_ESM.docx]
